# Micron-scale geometrical features of microtubules as regulators of microtubule organization

**DOI:** 10.7554/eLife.63880

**Published:** 2021-06-11

**Authors:** Nandini Mani, Sithara S Wijeratne, Radhika Subramanian

**Affiliations:** 1 Department of Molecular Biology, Massachusetts General HospitalBostonUnited States; 2 Department of Genetics, Harvard Medical SchoolBostonUnited States; Utrecht UniversityNetherlands; Utrecht UniversityNetherlands

**Keywords:** microtubule, self-organization, microtubule-associated proteins (MAPs), motor proteins, cytoskeleton, geometry, cellular architectures, micron-scale cellular structures

## Abstract

The organization of micron-sized, multi-microtubule arrays from individual microtubules is essential for diverse cellular functions. The microtubule polymer is largely viewed as a passive building block during the organization process. An exception is the ‘tubulin code’ where alterations to tubulin at the amino acid level can influence the activity of microtubule-associated proteins. Recent studies reveal that micron-scale geometrical features of individual microtubules and polymer networks, such as microtubule length, overlap length, contact angle, and lattice defects, can also regulate the activity of microtubule-associated proteins and modulate polymer dynamics. We discuss how the interplay between such geometrical properties of the microtubule lattice and the activity of associated proteins direct multiple aspects of array organization, from microtubule nucleation and coalignment to specification of array dimensions and remodeling of dynamic networks. The mechanisms reviewed here highlight micron-sized features of microtubules as critical parameters to be routinely investigated in the study of microtubule self-organization.

## Introduction

Self-organization is a recurrent theme in biology, evident across multiple length scales ranging from the oscillations of nanometer-sized signaling molecules in the cytoplasm to the formation of micron-sized cellular organelles and the patterning of millimeter-sized tissues. One of the best cellular systems for elucidating the principles of self-organization is the microtubule cytoskeleton, where dynamic microtubule polymers are organized into micron-sized arrays (Karsenti, 2008). Every microtubule filament within a multi-microtubule array is a polymer of tubulin subunits, polymerized with a specific polarity. Despite being assembled from the same microtubule building block, microtubule-based arrays display considerable diversity in their overall dimensions, shape, and polarity, in a manner that is intricately linked to their function. For instance, the orientation of coaligned, overlapping microtubules in the cortex of plant cells specifies the axis of cell elongation, and the polarity of long microtubule bundles in neurons distinguishes axons from dendrites (Chen et al., 2016; Kelliher et al., 2019). Understanding the mechanisms that direct the formation of distinct cellular arrays and specify their architecture will bring to light the general design principles underlying self-organization.

Building a multi-microtubule array of defined architecture requires the regulation of dimensions of individual polymers, as well as organizing them into higher-order structures. Microtubules are characterized by their inherent dynamic instability and cycle between phases of polymerization and depolymerization (Mitchison and Kirschner, 1984). The parameters governing dynamic instability therefore specify the stability, length, and density of microtubules within an array. There are broadly two ways in which dynamic instability is thought to be regulated. First, polymerization dynamics of individual microtubule filaments are sensitive to the concentration of soluble tubulin heterodimers which constitute their building blocks (Walker et al., 1988). In cells, tubulin levels are in turn autoregulated through translational control by tubulin itself (Cleveland, 1988; Lin et al., 2020). Second, the dynamics of microtubule polymers are regulated by the activity of microtubule-associated proteins (MAPs), which include both motor and non-motor proteins. In addition to polymer length and stability, MAPs also mediate the organization of microtubules into arrays through activities such as lattice severing, cross-linking, and sliding of microtubule pairs (Watanabe et al., 2005; Subramanian and Kapoor, 2012; Gardiner, 2013; Kapitein and Hoogenraad, 2015; Bodakuntla et al., 2019). For these reasons, tubulin heterodimers and MAPs have traditionally been the focal point in understanding the mechanisms that determine the assembly and overall architecture of an array. Microtubules themselves are largely viewed as passive building blocks in this process. A notable exception is the ‘tubulin code’, which is generated through the diversity in tubulin isotypes and post-translational modifications on the microtubule lattice. These chemical modifications of tubulin at the amino acid level can affect the inherent physical properties of the polymer and also regulate MAP activity (Alper et al., 2014; Kaul et al., 2014; Sirajuddin et al., 2014; Leo et al., 2015; Valenstein and Roll-Mecak, 2016; Bailey et al., 2015; Schwarz et al., 1998; Robison et al., 2016; Portran et al., 2017). An in-depth discussion of the tubulin code hypothesis can be found in several excellent reviews and will not be discussed further (Janke and Kneussel, 2010; Song and Brady, 2015; Gadadhar et al., 2017; Wloga et al., 2017; Janke and Magiera, 2020; Verhey and Gaertig, 2007). In recent years, an exciting concept that has emerged is that microtubules can encode information on the micron-scale to direct the self-organization of arrays. The features of the microtubule lattice that provide this information fall into two categories: (i) structural defects on the microtubule lattice such as missing tubulin subunits or changes in protofilament arrangement and (ii) size and relative arrangement of polymers, specified by parameters such as microtubule length, and the overlap length or contact angle between two adjacent polymers. Together, these micron-scale geometrical features along with the nanometer-scale tubulin code modulate the dynamics of individual polymers and regulate the activity of associated MAPs. In the following sections, we discuss how regulation by geometrical features of lattices is central to several processes involved in building a multi-microtubule array and determining its architecture. Overall, these mechanisms highlight the role of microtubules - as master architects directing their assembly and organization into cellular arrays.

## How geometrical features of microtubules direct array organization

### Microtubule-templated microtubule assembly

‘I don't see how he can ever finish, if he doesn't begin.’
– Alice’s Adventures in Wonderland, Lewis Carroll

Polymerization of microtubules begins with nucleation (Roostalu and Surrey, 2017). In cells, microtubules are nucleated and anchored at specialized organelles such as centrosomes and spindle pole bodies that serve as microtubule-organizing centers (MTOCs) (Wu and Akhmanova, 2017; Paz and Lüders, 2018). It is now apparent that not all microtubules are nucleated from conventional MTOCs (Petry and Vale, 2015; Basnet et al., 2018; Yi and Goshima, 2018; Lüders, 2021). For example, nucleation has been observed on ‘acentrosomal’ sites which include chromatin, nuclear membranes and golgi in animal cells, and plastids and plasma membranes in plant cells (Lee and Liu, 2019; Lüders and Stearns, 2007). A well-studied non-MTOC-based mechanism is branching microtubule nucleation, where new microtubules are generated from the lattice of a pre-existing ‘parent’ microtubule.

**Figure 1.**
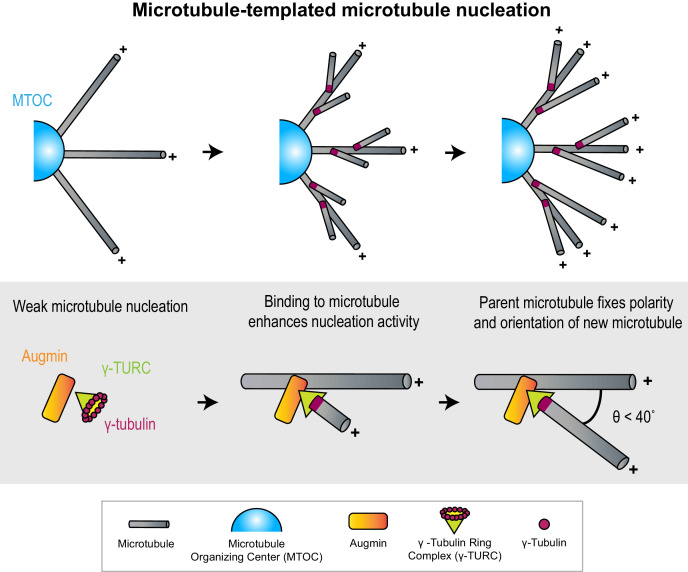
Microtubules act as micron-scale platforms for the generation of new microtubules in an array. *Top*: Branching microtubule nucleation increases the density of microtubules growing out from a microtubule organizing center (blue hemisphere), while preserving array polarity. *Bottom*: The nucleation activity of γ-tubulin ring complex (green cone) and augmin (orange rectangle) is enhanced by their recruitment to a microtubule lattice. The conformation of the ternary complex specifies the orientation (θ) and polarity (+) of the new microtubule with respect to the parent.

Since its first report in the alga *Nitella tasmanica*, branching nucleation has been observed to generate new microtubules at distinct orientation to the parent tubule in diverse cell types (O. WASTENEYS and E. WILLIAMSON, 1989). For example, newly nucleated microtubules are parallel to the parent in the spindle and neuronal axons, anti-parallel in arrays in yeast interphase cells and *Arabidopsis* pavement cells, and oriented at an angle of 40° with respect to the parent microtubule in plant cortical arrays (Janson et al., 2005; Murata et al., 2005; Petry et al., 2013; Sánchez-Huertas et al., 2016; Yagi et al., 2018). In this section, we discuss how pre-existing microtubules together with the γ-tubulin ring complex (γ-TuRC) direct the generation of new microtubules, and influence the polarity, orientation, and density of microtubules within an array (Figure 1).

#### Microtubules as micron-scale nucleation platforms

γ-TuRC is a cone-shaped protein assembly, over 2 MDa in size (Zheng et al., 1995; Oegema et al., 1999; Murphy et al., 2001; Liu et al., 2021). It contains a ring of γ-tubulins, which serves as an initiation point for the assembly of α-β tubulin subunits into a cylindrical microtubule (Kollman et al., 2008; Consolati et al., 2020; Liu et al., 2020; Wieczorek et al., 2020). To initiate branching nucleation, γ-TuRC must first be recruited to pre-existing microtubules through interaction with lattice-bound MAPs such as augmin and targeting protein for Xklp2 (TPX2) (Goshima et al., 2008; Petry et al., 2013; Liu et al., 2014; King and Petry, 2020). Notably, neither augmin nor TPX2 enhances the nucleation activity of γ-TuRC in solution, but do so when bound to a microtubule lattice, thus providing a route to microtubule nucleation on a pre-existing microtubule platform (Nakamura et al., 2010; Alfaro-Aco et al., 2020; Consolati et al., 2020).

#### Growth and polarity determination by parent microtubule

The inherent polarity of the parent microtubule can define the polarity of an array. Structural models for the ternary complex of augmin and γ-TuRC bound to the microtubule lattice predict that the stereospecificity of the bound γ-TuRC complex can restrict the angle and polarity of the newly nucleated microtubule with respect to the parent, thereby specifying array architecture (Wieczorek et al., 2020; Song et al., 2018). In support of the structural models, knock-outs and mutations of γ-TuRC subunits were seen to affect the distribution of microtubule branching angles in plant cells (Kong et al., 2010; Nakamura and Hashimoto, 2009).

#### Density determination by branching nucleation

How is the number of newly generated microtubules controlled by pre-existing microtubules? Multiple in vitro studies show that the nucleation sites for new microtubules are distributed all along the parent lattice, with no spatial bias toward either end (Song et al., 2018; Alfaro-Aco et al., 2020; Tariq et al., 2020). This suggests that the length of the parent microtubule could modulate microtubule number in an array, with more branching sites on longer microtubules.

In addition to γ-TuRC, branching microtubule nucleation can also be mediated by the Sjögren’s syndrome nuclear autoantigen 1 (SSNA1) protein. Interestingly, in addition to promoting branching nucleation in a similar manner to γ-TuRC, SSNA1 is observed to induce branching by splitting the microtubule lattice, in vitro. Subsequent tubulin polymerization on the splayed protofilaments can complete the cylindrical tubules to form a branched microtubule array (Basnet et al., 2018). Another special case of microtubule-dependent nucleation is the formation of doublet and triplet microtubules which are found in centrioles and in the axonemes of cilia and flagella. Here, ‘partial tubules’ which form incomplete rings in cross-section are assembled along the length of a complete tubule. In vitro work suggests that tubulin-tubulin interactions on the lattice of the parent microtubule can direct the assembly of the partial tubules that make up doublets and triplets (Serrano et al., 1984; Schmidt-Cernohorska et al., 2019).

What are the advantages of branching nucleation over nucleation from conventional MTOCs? First, it is predicted that radial outgrowth from an MTOC leads to a reduction in microtubule density with increasing distance from the MTOC (Mitchison et al., 2012). Branching nucleation on these initial radial microtubules helps maintain microtubule density at sites that are distant from the MTOC (Figure 1). Moreover, the kinetics of nucleation and the directional bias imparted to microtubules nucleated by this mechanism is suited for relatively rapid assembly of parallel bundles of high density, as seen in kinetochore fibers during cell division (David et al., 2019). Second, not all cells contain conventional MTOCs. For instance, in plant cells which lack centrosomes, branching nucleation is responsible for the genesis of a significant proportion of microtubules (Tian and Kong, 2019). In fact, it accounts for over 90% of microtubules found in *Arabidopsis* cortical arrays and is also important in post-mitotic neurons after the centrosome is inactivated (Cunha-Ferreira et al., 2018; Nakamura et al., 2010).

In summary, these examples illustrate distinct mechanistic routes to generate new microtubules from pre-existing filaments. Microtubules can serve as platforms to generate new tubules by activating the nucleation machinery, microtubule protofilaments can be splayed apart to serve as templates for new microtubules, and partial tubules can be assembled on a microtubule lattice to give rise to structures such as doublets. In these mechanisms, the linear and polar form of polymeric tubulin facilitates the determination of orientation, polarity, and density of new microtubules.

## Contact angle-based microtubule coalignment in arrays

‘Would you tell me, please, which way I ought to go from here?’
‘That depends a good deal on where you want to get to’
– Alice’s Adventures in Wonderland, Lewis Carroll

**Figure 2.**
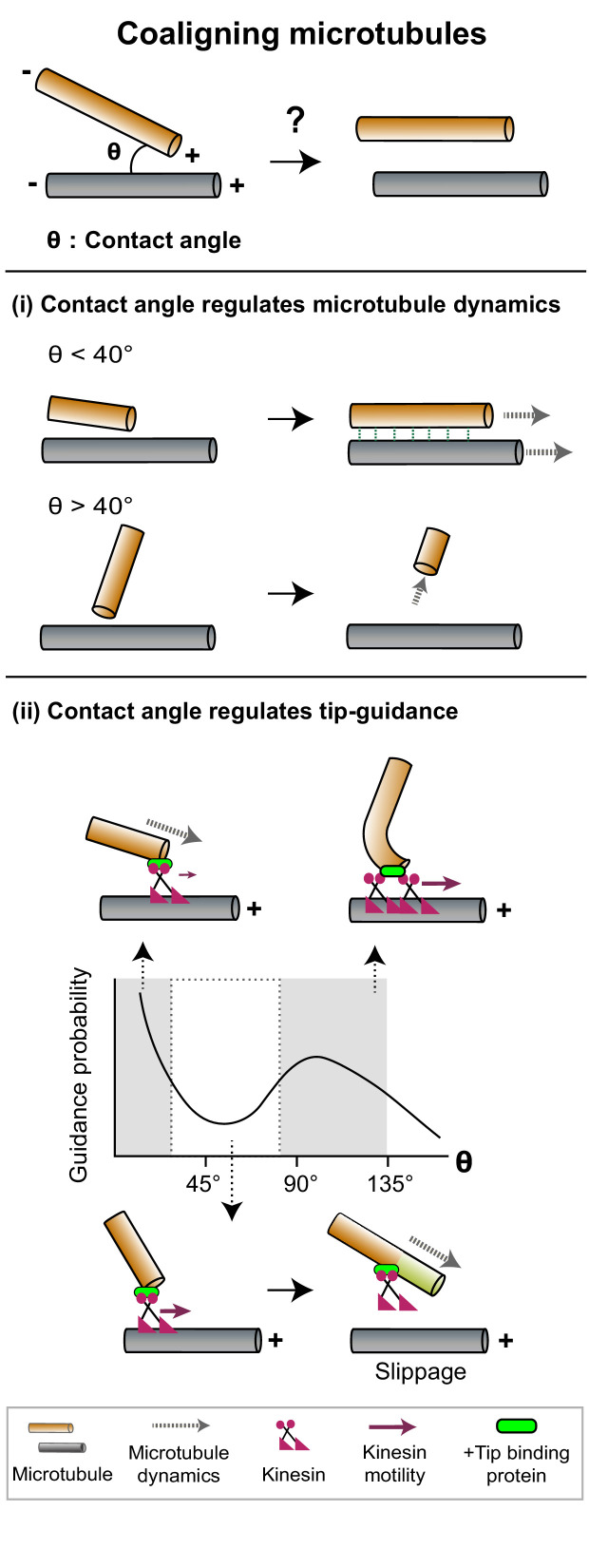
The contact angle between two microtubules regulates mechanisms that promote filament coalignment. *Top*: The contact angle (θ), defined as the angle between lines joining the point of contact between two microtubules and their respective minus ends, is an important geometrical feature that regulates microtubule coalignment. (**i**) A microtubule tip that encounters a lattice at a shallow angle (θ < 40°) gets cross-linked with the lattice and continues growing (top), while a microtubule that makes a steep angle (θ > 40°) undergoes collision-induced catastrophe (bottom). (**ii**) The probability of occurrence of microtubule tip-guiding events is determined by the contact angle (θ). *Top*: Tip guiding is mediated by motile kinesins (magenta) and +tip binding proteins (green). Guiding occurs through polymer bending at high contact angles (θ > 85°). *Bottom*: At intermediate angles (40° < θ < 85°), the need for continuous MAP occupancy of the newly polymerized tip (light green lattice) causes slippage events which decrease guidance probability.

Microtubules in cells are organized into specialized arrays. Upon close inspection, arrays are seen to contain recurring motifs or arrangements of microtubules A frequently observed motif is one where two polymers are coaligned. This can result in networks where adjacent microtubules are parallel to each other such as in ciliary axonemes and neuronal axons, anti-parallel as in the midzone of the mitotic spindle, or form arrays of mixed polarity such as plant cortical arrays (Subramanian and Kapoor, 2012). In some cases, these aligned arrays emerge from microtubules that are initially randomly oriented relative to each other. How are randomly oriented microtubules coaligned? Several studies highlight the importance of a geometrical parameter, the contact angle, as an important determinant in mechanisms promoting microtubule coalignment. For clarity, we define the contact angle as the angle between the lines joining the point where two microtubules encounter each other and their respective minus-ends (Figure 2).

### Contact angle regulates microtubule dynamics

Plant cortical arrays are characterized by the coalignment of microtubules with mixed polarity (Dixit et al., 2006; Wasteneys and Ambrose, 2009). These microtubules are nucleated from dispersed sites in the cortex and are initially oriented randomly to each other. To form the cortical array, longitudinally aligned microtubules need to be stabilized, and ‘discordant’ microtubules that grow at steep contact angles to the longitudinal array need to be removed. Dixit and Cyr, 2004 proposed that the contact angle a growing microtubule makes when it encounters another microtubule can determine its stability, to specify its eventual fate. Microtubules that intersect at shallow contact angles (<40°) are selectively cross-linked by MAPs such as members of the MAP65 family to form coaligned bundles. While the individual filaments within a bundle may be dynamic and exhibit tubulin turnover, the bundle as a whole has higher positional stability arising from the increased likelihood that at least one of its filaments is present at a given position (Wasteneys and Ambrose, 2009; Wightman and Turner, 2007; Ehrhardt and Shaw, 2006). In contrast, a steep contact angle (>40°) between a growing end and a pre-existing microtubule lattice generates a pushing force on the growing tip (Chi and Ambrose, 2016). Such forces can induce catastrophes, likely through a reduction in the rate of tubulin addition at plus-ends (Janson et al., 2003). Another consequence of the increase in contact angle is the altered accessibility of MAPs (Deinum et al., 2017). Together, force-induced catastrophe and differential MAP accessibility lead to the destabilization and eventual loss of discordant microtubules (Figure 2(i)). Thus, the selective modification of microtubule dynamics specified by a simple geometric feature such as contact angle between microtubules can generate an array with coaligned polymers.

### Contact angle regulates microtubule tip guiding

The function of certain arrays relies on coaligned microtubules with specific polarity, such as parallel microtubule arrays in epithelial cells and invertebrate neurons (Bacallao et al., 1989; Stone et al., 2008). A mechanism that can align two microtubules parallel to each other is microtubule tip guidance or steering. Here, coalignment occurs through the bending and guiding of a growing microtubule tip along the lattice of a microtubule it encounters (hereafter referred to as the rail), by motor proteins. For example, in branched neurons, the interaction between the motor kinesin-2 and the plus-tip tracking proteins adenomatous polyposis coli (APC) and end-binding protein 1 (EB1) promote the parallel alignment of a growing microtubule with a pre-existing bundle following the point of encounter (Mattie et al., 2010).

Reconstitution of tip guidance starting from randomly oriented dynamic microtubules and chimeric kinesins modified to bind to the plus-tip tracking EB1 have revealed that the probability of coalignment depends on the microtubule-binding affinities and tip occupancy of the MAPs (Chen et al., 2014). The occurrence of tip guidance events also depends on the contact angle between the two microtubules (Figure 2(ii)). Tip guidance is found to be more probable at very low angles and close to 90°, but less so at intermediate (40°−85°) and high obtuse angles (Doodhi et al., 2014). Modeling the microtubule as a rod bending under load predicts that the forces required for bending microtubules would permit guidance at low angles and limit it at high obtuse angles. However, it does not explain the decrease in frequency of guiding at intermediate angles. Experiments reveal frequent slippage of the growing microtubule along the rail in the intermediate angle regime as a probable mechanism. Slippage occurs because steering a tip along a rail at intermediate contact angles requires both tubulin polymerization and kinesin-mediated guiding at the tip. The need for continuous kinesin occupancy at the newly grown tip, in order to maintain contact with the rail, thus limits effective tip guiding. Slippage is not a limiting factor at higher angles where guidance is dominated by polymer bending (Figure 2(ii)).

The examples in this section illustrate that the encounter angle is a key variable that regulates polymer dynamics, MAP accessibility, and cross-linking probability. Thus, the initial orientation of polymers and the angles at which growing microtubules intersect one another are important determinants of structural features such as the organization and polarity of microtubules in the final array.

## Length and polarity-dependent regulation of MAP activity

‘Oh, I'm not particular as to size, only one doesn't like changing so often, you know’
– Alice’s Adventures in Wonderland, Lewis Carroll

An army of diverse Microtubule Associated Proteins MAPs serve as ‘builders’ to determine the size and shape of arrays through functions such as regulating microtubule dynamics, severing, cross-linking, and relative sliding between microtubules (van de Willige et al., 2016; Elliott and Shaw, 2018; Conkar and Firat-Karalar, 2021). The activity of MAPs, in turn, is found to be modulated by the geometrical parameters that specify array dimensions. On a single microtubule, the relevant parameter is polymer length. For pairs of microtubules, additional geometrical parameters that arise include the relative polarity, ‘overlap length’ over which two adjacent microtubules are cross-linked, the ‘overlap width’, which is the perpendicular distance between their lattices, and contact angle at their site of encounter. In the following section, we discuss the mechanisms by which these geometrical features regulate MAPs, and highlight how the interplay between geometrical parameters and MAP activity sculpt cellular arrays.

### Part 1: Microtubule length as a regulator of microtubule dynamic instability

**Figure 3.**
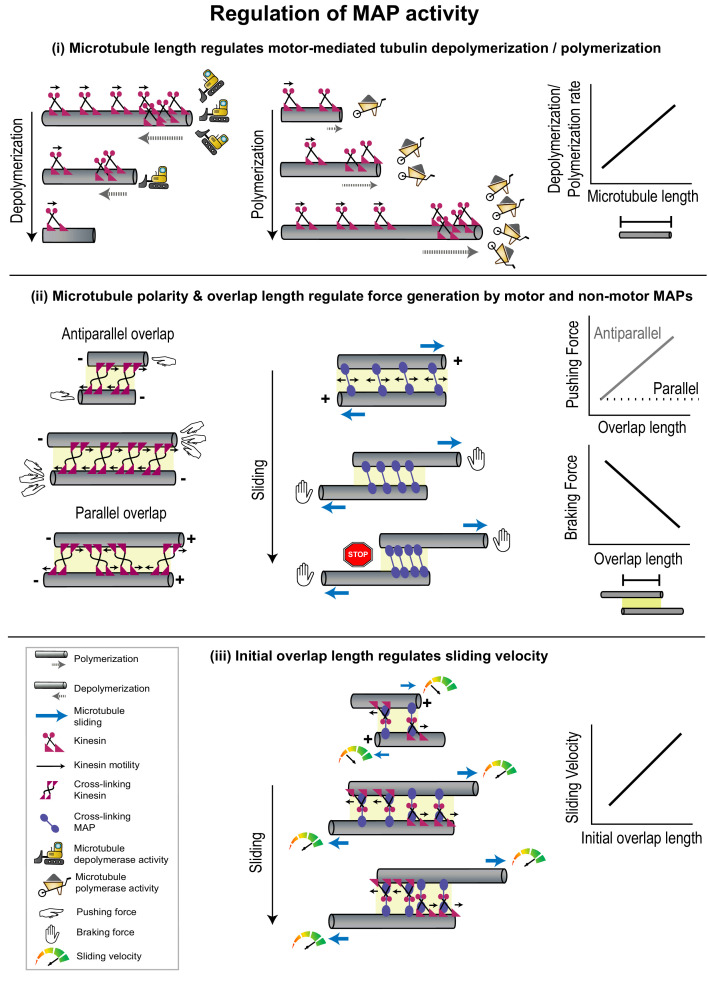
Geometrical features of individual microtubules or polymer networks regulate MAP activity. (**i**) The rate of microtubule depolymerization (left) and polymerization (middle) mediated by kinesin motors (magenta) is regulated by microtubule length (right) which determines the number of motors that accumulate at the plus-end. The change in length during depolymerization and polymerization provides feedback for further motor activity. (**ii**) Pushing forces (left) produced by motors (magenta) and braking forces (middle) produced by non-motor MAPs (violet) are regulated by the relative polarity (+/-) and overlap length (yellow shaded box) of cross-linked microtubules (right). Forces generated by individual cross-linking motors in an anti-parallel overlap are integrated to produce the net pushing force (left). Entropic expansion forces generated by non-motor MAPs increase when the overlap shrinks and MAP density in the overlap increases, to resist motor-mediated sliding (middle). (**iii**) The initial sliding velocity (red to green speedometer) in a system consisting of microtubules loosely coupled to each other by a motor protein (magenta) bound to a microtubule cross-linker (violet) is determined by initial overlap length and does not change as the overlap shrinks and cross-linker density increases.

The lengths of individual microtubules are determined by protein regulators of microtubule dynamic instability (Howard and Hyman, 2007). Several studies have uncovered the existence of feedback loops between regulator activity and microtubule length. For example, in budding yeast astral and kinetochore microtubules, the rate of tubulin disassembly by the kinesin-5 motor Cin8p increases with microtubule length (Gardner et al., 2008). Similarly in vitro studies reveal that the rate of microtubule depolymerization by kinesin-8 family members Kip3p and Kif18A and the rate of microtubule polymerization by the budding yeast kinesin Kip2p are proportional to microtubule length (Figure 3(i); Gupta et al., 2006; Varga et al., 2006; Mayr et al., 2007; Varga et al., 2009; Hibbel et al., 2015).

The microtubule length-dependent regulation of microtubule dynamics has been best studied in the context of Kip3p and explained by a mechanism called the ‘antenna model’ (Varga et al., 2006). Here, motor molecules land stochastically all along the lattice and step toward microtubule plus-ends. For highly processive motors with a slow dissociation rate from the microtubule end, such as in the case of Kip3p, the number of motors accumulated on the plus-ends is proportional to microtubule length (Figure 3(i)). Consequently, the rate of polymerization or depolymerization at the tip, which is mediated by motors, scales with microtubule length. An increase in depolymerization activity at the plus-end shortens microtubules, thereby providing negative feedback by reducing further motor accumulation and depolymerization rates. The antenna model thus illuminates the biophysical principles by which highly processive motors or motor complexes can alter microtubule dynamics in a microtubule length-dependent manner [(Subramanian et al., 2013; Leduc et al., 2012 Varga et al., 2006).].

### Part 2: Relative filament polarity regulates motor activity

#### Relative microtubule polarity regulates direction of motor stepping

For a pair of cross-linked microtubules, the relative orientation of the filaments has been remarkably found to alter the directionality of kinesin motors stepping in some instances. Among kinesin-5 proteins, the budding yeast Cin8p motor switches from minus-end directed on single microtubules to a plus-end directed motor on anti-parallel overlaps (Kapitein et al., 2008; [Bibr bib47][Bibr bib131]). While the precise molecular mechanism is under investigation, the change in Cin8p directionality is proposed to arise from the mechanical coupling of teams of motors via the microtubules they cross-link. A switch in motor directionality has also been observed for the minus-end directed kinesin-14 motor Cik1-Kar3 from budding yeast. When a lattice-bound Cik1-Kar3 motor is coupled to a second dynamic microtubule via interaction with an EB1 homolog, the motor switches to plus-end directed movement. Here, the force generated by the polymerization of the microtubule at its growing tip is proposed to be relayed via EB1 to the kinesin, resulting in reversal of motor direction (Molodtsov et al., 2016).

#### Relative microtubule polarity regulates force generation by motors

Within a microtubule array, cross-linking motors can generate forces to slide one microtubule with respect to another, thus varying the overlap length as well as the end-to-end length of an array (Forth and Kapoor, 2017; Vukušić et al., 2019). Direct force measurements on pairs of microtubules cross-linked by *Xenopus laevis* kinesin-5 motors have revealed that sliding forces produced by the motor depend on the relative polarities of the microtubules they cross-link (Shimamoto et al., 2015). On overlaps formed by anti-parallel microtubules, sliding forces were found to scale linearly with motor number whereas on parallel overlaps they were bidirectional, smaller in magnitude, and independent of motor number (Figure 3(ii)). The dependence of these sliding forces on microtubule polarity arises from the stochastic stepping of motors toward plus-ends. The force generated by one motor is transmitted via the microtubule and distributed to all other motors in the overlap. On anti-parallel overlaps this allows all motors to adjust their stepping velocity to maximize their force production and leads to a linear integration of forces generated by each motor. On parallel microtubules, however, the integration of forces produced by stochastically stepping motors leads to a fluctuating force output. Thus, the regulation of kinesin-5-mediated force output does not occur through the modulation of the mechanochemical cycle of the motor, but instead by the regulation of the total force applied by an ensemble of motor molecules in response to geometrical features of cross-linked microtubules. This mode of regulation can be advantageous in a structure like the mitotic spindle, where kinesin-5 motors cross-link both the parallel bundles found near the spindle poles and anti-parallel bundles at the cell equator (Shimamoto et al., 2015; Leary et al., 2019).

### Part 3: Overlap length-dependent regulation of forces generated by MAPs

#### Microtubule overlap lengths regulate forces by MAPs

The overlap length between two microtubules can determine the total number of force-generating cross-linking proteins. As described in the previous paragraph, sliding forces scale with overlap length due to the integration of force generated by individual kinesin-5 motors (Figure 3(ii); Shimamoto et al., 2015). Such overlap length-dependent force generation has been observed not only for motors which produce forces that assist sliding, but also for resistive or braking forces generated by non-motor cross-linkers, which prevent the complete separation of sliding microtubules (She et al., 2019; Gaska et al., 2020; Pringle et al., 2013; Stanhope et al., 2017).

Anaphase spindle elongation (Ase1p) is a non-motor protein which preferentially cross-links anti-parallel microtubules in fission yeast interphase arrays and the mitotic spindle midzone (Janson et al., 2007). In vitro studies have shown that Ase1p resists motor forces by producing a braking force that increases as motor-mediated sliding progresses (Figure 3(ii); Janson et al., 2007; Braun et al., 2011). Mechanistically, this has been explained by drawing an analogy to confined gas molecules which exert entropic forces to minimize density by maximizing containment volume (Braun et al., 2011; Lera-Ramirez and Nédélec, 2019; Braun et al., 2016). In the absence of an external force, Ase1p molecules undergo one-dimensional diffusion within a microtubule overlap zone and can exert forces to minimize their density and maximize the overlap length. When Ase1p cross-linked microtubules are moved apart by motors, the overlap shrinks, resulting in an increase in Ase1p density, due to its low off-rate from overlaps (Figure 3(ii)). Consequently, the confined molecules exert entropic forces to maximize microtubule overlap and resist sliding. This forms an adaptive system, since the resistive force generated by Ase1p molecules increases as overlap length shrinks during sliding. The increase in resistive force eventually enables the formation of stable overlaps.

#### Microtubule overlap lengths regulate relative filament sliding velocity

In fission yeast, the rate of spindle elongation which is driven by the kinesin-mediated sliding of anti-parallel microtubules, is higher when the overlaps between microtubules are longer (Krüger et al., 2019). Such scaling of sliding velocity with overlap length or motor number is unusual for microtubule movement driven by an ensemble of processive kinesins (Howard et al., 1989). For example, in multi-motor surface gliding assays with conventional kinesin, microtubule movement velocity is independent of microtubule length (Hunt et al., 1994). This is due to the low viscous drag experienced by the moving microtubule in aqueous buffers and the high magnitude of forces generated by kinesin molecules. However, recent in vitro reconstitution experiments provide insights into two classes of mechanisms by which motor-mediated sliding velocity can scale with microtubule overlap length, even in the absence of substantial external viscous drag forces. The first class of mechanisms builds on the concept of increasing resistive entropic forces within shrinking overlap as described in the previous section. This phenomenon has been observed during microtubule sliding by the kinesin-14 HSET and by the combined activity of the kinesin-14 Ncd and the non-motor MAP Ase1p (Braun et al., 2011; Braun et al., 2017). A second mechanism harnesses the loose coupling between cross-linked microtubules for overlap length-dependent rate of sliding. This is illustrated in the case of sliding by the human Ase1p homolog, protein regulator of cytokinesis 1 (PRC1) and the kinesin Kif4A (Bieling et al., 2010; Subramanian et al., 2013; Wijeratne and Subramanian, 2018; Hannabuss et al., 2019). Here, the initial sliding velocity depends on the initial overlap length, but in contrast to the Ase1p-Ncd or HSET systems, does not change as overlap length reduces during sliding (Figure 3(iii)). In the PRC1-Kif4A system, cross-links that drive movement are likely formed by Kif4A motor domains on one microtubule and spectrin domains of PRC1 which are bound to the other microtubule, resulting in loose coupling between the two sliding microtubules. As a result, every 8 nm step taken by Kif4A on one microtubule does not translate to an 8 nm displacement of the other microtubule. Longer overlaps have more Kif4A and PRC1 molecules and therefore a higher probability of microtubule displacement per unit time (Figure 3(iii)). A similar mechanism is thought to drive length-dependent surface gliding by an ensemble of kinesin motors that are anchored to a diffusive lipid surface instead of a rigid glass coverslip (Grover et al., 2016).

When would the regulation of MAPs by geometrical features of microtubules be advantageous over other methods of controlling their activity? Structures such as the mitotic spindle are continuously remodeled on a timescale of minutes. Length-sensing mechanisms that provide ‘real-time’ feedback allow for adaptive restructuring of microtubule arrays, both during dynamic processes such as spindle assembly and elongation, and in response to localized perturbations like slippage or microtubule damage. These microtubule length-dependent mechanisms are distinct from mechanisms that involve the tubulin code, where real-time adaptation on the minutes timescale is more difficult due to the relatively slower rates of generating and removing post-translational modifications (Szyk et al., 2014; Szyk et al., 2011).

Together, these studies highlight that a defining feature of a polymer, its length, is an important variable in specifying array architecture. Altering microtubule length changes the total number or density of motor and non-motor MAPs on the lattice. In conjunction with the diversity in intrinsic MAP properties such as motor processivity, force generation, and microtubule dissociation rates, the microtubule length or the microtubule overlap length synergistically determines the dimensions and mechanical properties of cellular arrays. In addition, the micron-scale microtubule lattice can couple ensembles of MAPs, leading to phenomena as drastic as the reversal of stepping direction of teams of motors in response to relative filament polarity.

## Lattice defects as regulators of microtubule dynamics

‘I know who I was when I got up this morning, but I think I must have been changed several times since then.’
– Alice’s Adventures in Wonderland, Lewis Carroll

In the crowded cellular volume, growing microtubules encounter physical obstacles such as other cytoskeletal arrays, cell membranes, and chromosomes, which can cause the filaments to undergo bending, buckling, or bundling (Odde et al., 1999; Waterman-Storer and Salmon, 1997). The stepping of motor proteins and the action of severing enzymes can also physically damage the lattice (Srayko et al., 2006; Dumont et al., 2015). As a result, both in vitro and in cells, microtubules display ‘lattice defects’, which are disruptions in the ordered arrangement of tubulin subunits in a polymer, extending over length scales ranging from 0.1 to 1 μm (Chrétien et al., 1992; Atherton et al., 2018). These defects manifest as vacancies in the lattice caused by missing tubulin heterodimers, sharp edges on the tubule due to abrupt shifts in protofilament number, or as regions of structural heterogeneity. Despite being localized to discrete sites along the microtubule lattice, defects have been shown to affect plus-end dynamics. For instance, a mismatch in protofilament number on the lattice can lead to an increase in catastrophe frequency (Rai et al., 2021). Sites of microtubule damage can also recruit MAPs whose activity changes the total density of microtubules in cellular arrays through the following known mechanisms.

### Lattice repair increases microtubule lifetimes

**Figure 4.**
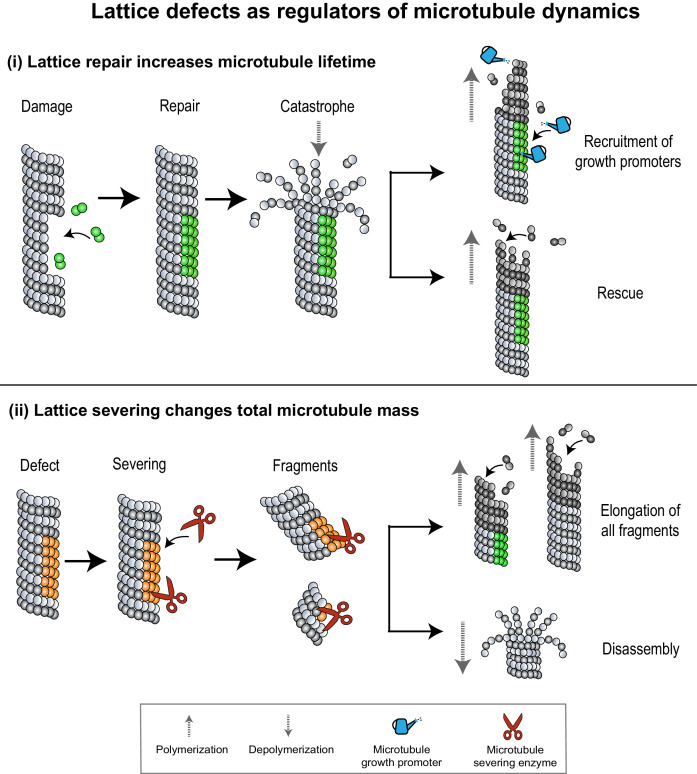
Lattice defects can help recruit MAPs that regulate microtubule dynamics. (**i**) Sites of lattice damage can be repaired through the incorporation of GTP-tubulin (green spheres) from solution. These GTP-islands on the lattice help increase microtubule lengths and lifetimes by recruiting microtubule growth promoters (blue watering can) or by serving as points of rescue during subsequent catastrophe events. (**ii**) Lattice defects (orange spheres) can recruit microtubule severing enzymes (red scissors) to change microtubule density in an array. Severed fragments can either increase total microtubule mass by serving as templates for further polymerization, or be disassembled.

Somewhat counterintuitively, studies in animal cells have shown that damage to microtubule lattices can result in an increase in the length and stability of microtubules (Aumeier et al., 2016). Several lines of experimental evidence indicate that sites of damage in the middle of a microtubule can incorporate GTP-tubulin dimers, as observed in live cells (Dimitrov et al., 2008; de Forges et al., 2016) and in microtubules polymerized in vitro (Schaedel et al., 2015; Schaedel et al., 2019; Rai et al., 2020). These ‘GTP-islands’ promote microtubule growth in two ways: (i) by serving as rescue points for re-initiation of growth during subsequent catastrophe events (Dimitrov et al., 2008; Tropini et al., 2012; Aumeier et al., 2016) and (ii) through the recruitment of growth-promoting MAPs that specifically recognize and bind to GTP-tubulin over GDP-tubulin, such as the rescue factors cytoplasmic linker protein of 170 kDa (CLIP-170) and CLIP-associating protein (CLASP) (Figure 4(i); Folker et al., 2005; de Forges et al., 2016; Aher et al., 2020). Consequently, as demonstrated by in vitro studies, sites of damage can result in microtubules that have significantly longer lifetimes, lengths, and rescue frequencies compared to microtubules undergoing stochastic polymerization and depolymerization (Aumeier et al., 2016). Thus, the repair of damaged lattices can infuse a fresh lease of life into a dynamic microtubule. This is advantageous in a crowded environment, where microtubules have a high probability of crossing over each other, which can result in lattice damage (de Forges et al., 2016). Repair at a site of damage increases the rescue frequency, resulting in an increase in the average length of polymers in the network. This further enhances the likelihood of microtubule crossovers, lattice damage, and rescue, thus increasing the overall density and lifetime of microtubules in the array.

### Lattice severing changes the total microtubule mass

Pre-existing microtubules can change the total microtubule number by recruiting severing enzymes which catalyze the removal of tubulin subunits to break the microtubule into shorter polymers (McNally and Roll-Mecak, 2018). Some severing enzymes like katanin have been predicted to preferentially bind to sites of lattice damage on microtubules, based on in vitro experiments and simulations (Davis et al., 2002; Díaz-Valencia et al., 2011). Severing activity can counterintuitively also lead to an increase in the overall microtubule mass (Figure 4(ii); Roll-Mecak and Vale, 2006; Srayko et al., 2006). For example, in *Caenorhabditis elegans* meiotic spindles, the severing of microtubules by katanin leads to a steep decrease in microtubule density near the poles and an increase in microtubule density at the cell center (Srayko et al., 2006; McNally et al., 2006). It is proposed that short microtubules generated by severing are redistributed to the cell center and organized into a bipolar array. Curiously, instead of conserving the total microtubule mass, the activity of katanin in this system was found to cause an increase in the overall cellular microtubule mass. In vitro studies on the severing enzymes katanin and spastin have provided an explanation, showing that the activity of severing enzymes leads to the removal of tubulin dimers and causes lattice damage (Vemu et al., 2018; Kuo et al., 2019). Repair of these sites through incorporation of GTP-tubulin dimers from solution leads to an increase in total microtubule mass and number through a combination of two mechanisms: (i) When the rate of GTP-tubulin incorporation exceeds rate of enzyme-catalyzed tubulin removal, the lattice acquires GTP-islands which increase rescue probability and decrease shrinkage rates during subsequent depolymerization events. (ii) When the rate of tubulin removal is higher than tubulin addition, enzyme activity severs the lattice. However, the newly created plus-ends contain GTP-tubulin dimers which increase their stability, and polymerization can be re-initiated from the severed microtubule ends. In vitro, it has also been shown that soluble tubulin itself can bind katanin and inhibit its activity, thus setting up a feedback loop for the control of microtubule length and density (Bailey et al., 2015).

Together, these examples show that the inter-dependence between structural features of microtubule lattices, such as defects, and the activity of MAPs including severing enzymes and rescue factors can modulate polymer length and dynamics. This interplay serves to change microtubule density in an array through the disassembly, redistribution, stabilization, or amplification of microtubules.

## Integration of microtubule-based mechanisms in cells

‘Begin at the beginning,’ the King said gravely, ‘and go on till you come to the end: then stop.’
– Alice’s Adventures in Wonderland, Lewis Carroll

Microtubule arrays are dynamically assembled, remodeled, and disassembled over the lifetime of a cell. For example, the organization of the spindle constantly changes during cell division, plant cortical arrays are reoriented *en masse* to specify a new cell elongation axis in response to external stimuli, the polarities of microtubule bundles in neurons change as minor neurites grow into axons and dendrites, and the ciliary axoneme is built and disassembled each cell cycle (Sánchez and Dynlacht, 2016; van Beuningen and Hoogenraad, 2016). The assembly and remodeling of cellular arrays involves the integration of the various microtubule-regulated mechanisms discussed above such as microtubule nucleation, coalignment, MAP regulation, lattice severing, and repair. These processes do not occur in a discrete or sequential manner, but in fact occur concurrently, as illustrated by the two examples below.

### Reorganization of plant cortical arrays

**Figure 5.**
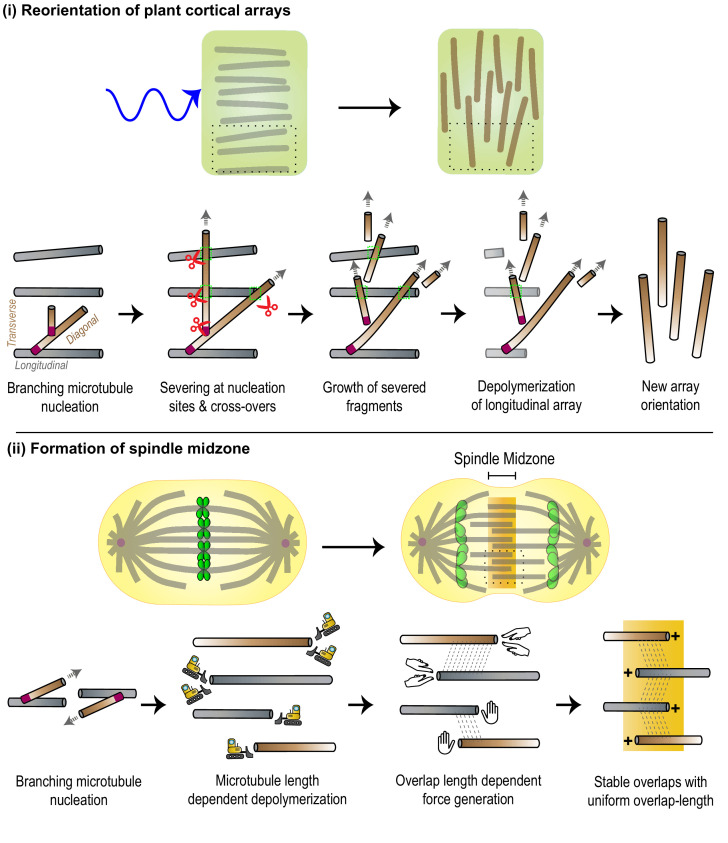
The integration of various microtubule-regulated mechanisms underlies the self-organization and remodeling of cellular arrays. (**i**) *Top*: Plant cortical arrays are reoriented by ~90° in response to blue light. *Bottom*: Zoomed-in view of region enclosed by black dashed box in top panel. Pre-existing microtubules (gray) of the longitudinal array generate new diagonal and transverse microtubules (brown) through successive branching microtubule nucleation events (magenta). New microtubules are amplified through severing (red scissors) at sites of nucleation and cross-overs (green dashed box), and polymerization of severed fragments. The disassembly of the original array completes the re-orientation process. (**ii**) *Top*: The spindle midzone, a cross-linked array of inter-digitating, anti-parallel microtubules is formed during anaphase to specify the site of cell cleavage and ensure error-free genome (green) propagation. *Bottom*: Zoomed-in view of region enclosed by black dashed box in top panel. Pre-existing microtubules (gray) serve as platforms to generate new parallel microtubules (brown) through branching microtubule nucleation (magenta). The location of the array at the center of the cell and its dimensions are specified by length-dependent microtubule depolymerization and generation of overlap length-dependent sliding and braking forces to produce stable overlaps.

Stimulation of plant cells by blue light results in a dramatic reorientation of their cortical microtubules by ~90°, to specify a new cell elongation axis (Zandomeni and Schopfer, 1993; Paredez et al., 2006; Chen et al., 2016). In this section, we refer to the initial array as the longitudinal array, and the final arrangement, which is perpendicular to the original array, as the transverse array (Figure 5(i)). The microtubules of the longitudinal array play a critical role in directing array reorientation (Lindeboom et al., 2013). The process begins with γ-TuRC-mediated branching microtubule nucleation, from the microtubules in the longitudinal array. This results in new microtubules which grow at angles close to ±40° (hereafter referred to as diagonal microtubules) to the longitudinal array. Subsequent branching nucleation from diagonal microtubules generates a subset of new microtubules that grow perpendicular to the longitudinal array and are oriented correctly to form the transverse array. However, to establish an array that is reoriented by 90°, the selective amplification of transverse microtubules and destabilization of longitudinal and diagonal microtubules is required. For this, the severing enzyme katanin is specifically recruited to sites where two microtubules cross over (Zhang et al., 2013; Deinum et al., 2017; Wang et al., 2017). At these sites, the frequency of katanin severing is higher on the new microtubule compared to microtubules of the original array, although the precise mechanism for selective severing is currently unknown. Katanin severing of a new microtubule generates two microtubules, doubling the number of transverse and diagonal microtubules that continue to grow (Nakamura et al., 2018). How does this mechanism preferentially amplify transverse over diagonal microtubules? A new microtubule forms the maximum number of cross-overs, when the distance between the pre-existing microtubules that it encounters is the least. Basic geometry dictates that this condition is satisfied when the new microtubule grows perpendicular to the original array, compared to all other orientations, thereby selectively amplifying the transverse microtubules. Once the transverse array is established, there is a reduction in branching nucleation rates by mechanisms such as phototropin signaling, and a reduction in severing at cross-overs by mechanisms that remain unclear. This halts both generation and amplification of new microtubules, and a stable transverse array is e. Thus, the longitudinal array serves as a patterned template for the preferential amplification of microtubules that grow at right angles to it, to form the transverse array (Figure 5(i)).

### Assembly of the mitotic spindle midzone

The spindle midzone is an inter-digitating array of anti-parallel microtubules at the cell center. (Mastronarde et al., 1993; Figure 5(ii)). The precise cellular location and size of the spindle midzone determine the site of cell cleavage during cytokinesis and ensure error-free genome propagation (Eggert et al., 2006; Scholey et al., 2016). The specification of both these parameters relies on several microtubule-regulated mechanisms. The formation of the spindle midzone first requires an increase in microtubule density at the cell center. For this, microtubules growing radially outward from the centrosomes serve as platforms for γ-TuRC and augmin-mediated branching nucleation resulting in new microtubules that grow with their plus-ends oriented toward the cell center (Uehara and Goshima, 2010; Uehara et al., 2016; Pamula et al., 2019). Here, they form overlaps with microtubules of the opposite polarity, through the action of cross-linking motors and non-motor MAPs. The location of this overlap must be maintained at the cell center even as the lengths of the constituent microtubules change as cell division progresses. The scaling of microtubule depolymerization rates with polymer length through the action of depolymerizing kinesins such as Kip3p is proposed to play an important role. Length-dependent depolymerization ensures that microtubules have uniform length, and overlaps between anti-parallel microtubules are formed at the center of the cell (Varga et al., 2006; Varga et al., 2009; Rizk et al., 2014). How is the overlap length of the array precisely specified? The initial overlap length between two microtubules is proposed to be a key determinant. This is because microtubule overlap length regulates microtubule polymerization dynamics and relative sliding of microtubules by several midzone kinesins. These include kinesin-5, kinesin-6, kinesin-14,, and the Kif4A-PRC1 complex (Glotzer, 2009; Bieling et al., 2010; Subramanian and Kapoor, 2012; Scholey et al., 2016; Wijeratne and Subramanian, 2018; Shimamoto et al., 2015; Hannabuss et al., 2019; Krüger et al., 2019). As sliding progresses, overlap length-dependent braking forces generated by motor and non-motor MAPs increase, while the rate of sliding decreases, to establish a stable midzone of defined length (Braun et al., 2011; Braun et al., 2017). In addition to the initial overlap length, relative microtubule polarity can also regulate sliding forces generated by midzone motors (Shimamoto et al., 2015). Thus, while the activity of MAPs alters the dimensions of the array, the changes in geometrical parametersthat define crosslinked microtubules in turn provide instantaneous feedback to regulate MAP activity. This interdependence between MAP activity and array geometry ensures that a stable spindle midzone array is formed at a fixed location in the cell, even as its constituent dynamic microtubules undergo growth, depolymerization, and relative sliding (Figure 5(ii)).

### Conclusion

**Figure 6.**
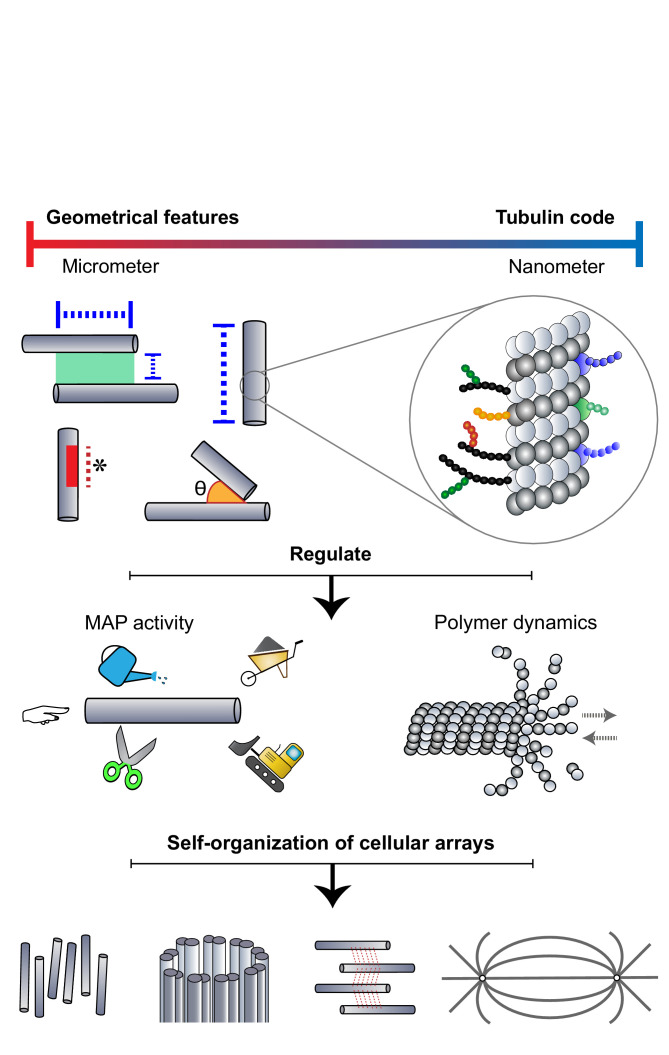
Microtubules adopt a DIY (Do It Yourself) approach toward array building. The microtubule lattice contains features at two length scales: micron-sized geometrical parameters encompassing the arrangement of polymers and structural defects of the lattice, and nanometer-scale "tubulin codes" comprising post-translational modifications and isotypes of tubulin. Both sets of features regulate MAP activity and polymer dynamics in order to direct the self-organization of microtubules into diverse cellular arrays.

The self-organization of microtubules into specialized micron-sized arrays has been extensively studied from the perspective of motor and non-motor MAPs that shape arrays. It is now evident that microtubules themselves provide cues to regulate MAP activity, both at the nanometer scale through a tubulin code and at the micron scale via geometrical features of the lattice. The interplay between microtubules and MAP activity directs the self-organization process, by specifying microtubule density, array architecture, and stability (Figure 6). This ‘DIY’ (Do It Yourself) approach adopted by microtubules toward their organization has far-reaching consequences in the cell. First, members of the different cytoskeletal families are known to engage in extensive crosstalk. (Dogterom and Koenderink, 2019; Logan and Menko, 2019; Seetharaman and Etienne-Manneville, 2020). For example, microtubules have been shown to regulate the nucleation, polymerization, and dynamics of the actin network in migrating cells, neuronal growth cones, and dendritic spines. In dividing cells, the position and geometry of the acto-myosin contractile ring at the cell center are determined by astral and spindle midzone microtubules (Pollard and O'Shaughnessy, 2019). Further, microtubule-based transport is also important for the assembly of intermediate filaments (Hookway et al., 2015). Second, microtubule networks are important in creating and maintaining biochemical concentration gradients in the cytosol. For instance, spindle microtubules are known to play a role in creating spatial gradients of Aurora B kinase activity and components of the Ran-GTP pathway (Noujaim et al., 2014; Oh et al., 2016). Thus, beyond directing the assembly of microtubules into arrays, micron-sized features of microtubule networks contribute to the larger-scale spatial organization of the eukaryotic cell by directing the organization of other cytoskeletal networks and signaling gradients.

Polymeric proteins that self-organize into micron-sized arrays are not exclusive to eukaryotic cells, but are found across all kingdoms of life. In bacteria and archaea, homologs of tubulin, actin, and intermediate filaments form filamentous assemblies, with the assistance of conserved proteins (Busiek and Margolin, 2015; Fink et al., 2016; Stairs and Ettema, 2020). Future studies will reveal if the mechanisms invoking the regulation of microtubule array assembly by geometrical features of microtubules could have emerged in prokaryotic ancestors. In addition to the assembly of protein-based structures, these principles may also apply to the self-organization of nucleic acids and other cellular polymeric arrays.

## Future outlook

- Multiple nucleation pathways co-exist in cells in addition to microtubule-templated microtubule nucleation, each yielding new filaments that grow at specific orientation and polarity with respect to their organizing centers. For instance, centrosome-nucleated microtubules grow out radially, golgi-nucleated microtubules are tangential to the golgi, and nuclear membrane-associated microtubules form longitudinal arrays (Miller et al., 2009; Lüders and Stearns, 2007; Musa et al., 2003). Whether there are specialized geometrical cues that dictate the organization of these arrays relative to pre-existing microtubule networks presents an exciting research direction.
- Several mechanisms facilitate the close packing of microtubules to form bundles, ranging from those that occur in the absence of all MAPs to others which utilize cross-linking proteins of varying lengths (Peterman and Scholey, 2009; Needleman et al., 2004b). Thus far, our understanding of the self-organization and function of microtubule bundles have largely come from studies on coaligned microtubules cross-linked by members of the Ase1p/PRC1 family and a few cross-linking motor proteins of the kinesin family. Physical mechanisms such as osmotic forces induced by macromolecules and formation of liquid crystalline domains also result in the bundling of microtubules (Hitt et al., 1990; Needleman et al., 2004a; Guo et al., 2008). Remarkably, the shape of microtubule bundles formed by these mechanisms and the motility of motor proteins within them are found to be different when compared to bundles formed with protein cross-linkers (Edozie et al., 2019; Conway et al., 2014). Whether the close packing of microtubules in the absence of all MAPs generates geometrical cues similar to those observed with protein cross-linkers is still under-explored. Extending this line of research to diverse protein and non-protein cross-linkers will expand our understanding of geometrical parameters in array organization.
- The role of geometrical parameters in the organization of microtubules into radial, polar arrays such as asters remains poorly understood (Needleman and Dogic, 2017; Rickman et al., 2019). Intriguingly, in vitro studies show that motor proteins belonging to the kinesin-5 or kinesin-14 families can promote either nematic (linear bundles) or polar (radial arrays) organization of microtubules, depending on motor velocity and number, soluble tubulin concentration, microtubule number, and growth rates (Roostalu et al., 2018; Norris et al., 2018). Whether geometric features of microtubules direct the formation of polar arrays by kinesin and dynein motors (Tan et al., 2018; Khetan et al., 2021), and mediate the switch between nematic and polar self-organization remains an open question.
- In addition to overlap length and intersection angles, a fundamental geometrical parameter that defines the relative placement of two microtubules within a close-packed network is the overlap width or the spacing between their lattices. The observation that cross-linking motors and MAPs are characterized by a range of molecular dimensions suggests that the overlap width parameter could (i) regulate selective access of MAPs to the microtubules in an array, which in turn can modulate bundle dynamics and structure (Conway et al., 2014), (ii) determine the 2D and 3D organization of polymers in an array, by varying the nearest-neighbor distances (Nixon et al., 2015; Needleman et al., 2004b). Reconstitution studies that systematically vary the overlap width are likely to provide more insights. The effect of overlap width and the size of the molecular cross-linkers is particularly interesting in the context of asters where the overlap width would vary along the lattice in a radial assembly of microtubules.
- Lattice defects such as bends and breaks in the microtubule wall have been shown in vitro to serve as entry points into the microtubule lumen for the enzyme αTAT1 where it acetylates tubulin on a residue facing the lumen (Coombes et al., 2016). This mechanism raises the exciting possibility that regions of missing tubulin subunits could serve as ‘sieves’, allowing for selective lumen entry of proteins based on size. In addition, a break in the ordered arrangement of tubulin subunits could also hinder motor motility. Precise measurements of the dimensions of lattice defects, including the number of tubulin dimers they span, number of protofilaments they extend over, and their effect on microtubule stability, motor motility, cross-linking, and severing probability will provide insights into how microtubule defects regulate microtubule organization.
- Microtubules serve as platforms to couple the activity of associated motors and non-motor MAPs. The best understood coupling mechanisms arise from short-range interactions such as steric effects and cooperativity or crowding (Vilfan et al., 2001; Leduc et al., 2012). Can the linear structure of microtubules mediate the coupling of MAP molecules over distances in the micron length scale? Prior literature raises the intriguing possibility that microtubules can act as a micron-scale coupling medium to influence microtubule-binding kinetics of MAPs and motile propertiesof motors (Muto et al., 2005; Wijeratne et al., 2020). For example, microtubule-bound kinesin-1 motors have been shown to increase the axial pitch of the microtubule lattice, an alteration that is thought to promote the cooperative binding of other kinesin-1 molecules (Peet et al., 2018; Shima et al., 2018). Similarly, the straightening of tips of protofilaments by EB1 is proposed to mediate the synergistic increase in microtubule growth rates in the presence of *Xenopus* microtubule-associated protein (XMAP215) and EB1 (Zanic et al., 2013). In addition to MAP binding and activity, long-range effects on the microtubule lattice can also modulate the dynamics of the polymer itself. For instance, the presence of a lattice defect site corresponding to protofilament number mismatch has been shown to affect the dynamics of the polymer’s plus-end which can be several microns away (Rai et al., 2021). Elucidating conditions for micron-scale coupling and uncovering new mechanisms by which the microtubule lattice facilitates long-range communication and allosteric regulation of MAPs promises to be an exciting new research direction.

## References

[bib1] Aher A, Rai D, Schaedel L, Gaillard J, John K, Liu Q, Altelaar M, Blanchoin L, Thery M, Akhmanova A (2020). CLASP mediates microtubule repair by restricting lattice damage and regulating tubulin incorporation. Current Biology.

[bib2] Alfaro-Aco R, Thawani A, Petry S (2020). Biochemical reconstitution of branching microtubule nucleation. eLife.

[bib3] Alper JD, Decker F, Agana B, Howard J (2014). The motility of axonemal dynein is regulated by the tubulin code. Biophysical Journal.

[bib4] Atherton J, Stouffer M, Francis F, Moores CA (2018). Microtubule architecture in vitro and in cells revealed by cryo-electron tomography. Acta Crystallographica Section D Structural Biology.

[bib5] Aumeier C, Schaedel L, Gaillard J, John K, Blanchoin L, Théry M (2016). Self-repair promotes microtubule rescue. Nature Cell Biology.

[bib6] Bacallao R, Antony C, Dotti C, Karsenti E, Stelzer EH, Simons K (1989). The subcellular organization of Madin-Darby canine kidney cells during the formation of a polarized epithelium. Journal of Cell Biology.

[bib7] Bailey ME, Sackett DL, Ross JL (2015). Katanin severing and binding microtubules are inhibited by tubulin carboxy tails. Biophysical Journal.

[bib8] Basnet N, Nedozralova H, Crevenna AH, Bodakuntla S, Schlichthaerle T, Taschner M, Cardone G, Janke C, Jungmann R, Magiera MM, Biertümpfel C, Mizuno N (2018). Direct induction of microtubule branching by microtubule nucleation factor SSNA1. Nature Cell Biology.

[bib9] Bieling P, Telley IA, Surrey T (2010). A minimal midzone protein module controls formation and length of antiparallel microtubule overlaps. Cell.

[bib10] Bodakuntla S, Jijumon AS, Villablanca C, Gonzalez-Billault C, Janke C (2019). Microtubule-Associated proteins: structuring the cytoskeleton. Trends in Cell Biology.

[bib11] Braun M, Lansky Z, Fink G, Ruhnow F, Diez S, Janson ME (2011). Adaptive braking by Ase1 prevents overlapping microtubules from sliding completely apart. Nature Cell Biology.

[bib12] Braun M, Lansky Z, Hilitski F, Dogic Z, Diez S (2016). Entropic forces drive contraction of cytoskeletal networks. BioEssays.

[bib13] Braun M, Lansky Z, Szuba A, Schwarz FW, Mitra A, Gao M, Lüdecke A, Ten Wolde PR, Diez S (2017). Changes in microtubule overlap length regulate kinesin-14-driven microtubule sliding. Nature Chemical Biology.

[bib14] Busiek KK, Margolin W (2015). Bacterial actin and tubulin homologs in cell growth and division. Current Biology.

[bib15] Chen Y, Rolls MM, Hancock WO (2014). An EB1-kinesin complex is sufficient to steer microtubule growth in vitro. Current Biology.

[bib16] Chen X, Wu S, Liu Z, Friml J (2016). Environmental and endogenous control of cortical microtubule orientation. Trends in Cell Biology.

[bib17] Chi Z, Ambrose C (2016). Microtubule encounter-based catastrophe in Arabidopsis cortical microtubule arrays. BMC Plant Biology.

[bib18] Chrétien D, Metoz F, Verde F, Karsenti E, Wade RH (1992). Lattice defects in microtubules: protofilament numbers vary within individual microtubules. Journal of Cell Biology.

[bib19] Cleveland DW (1988). Autoregulated instability of tubulin mRNAs: a novel eukaryotic regulatory mechanism. Trends in Biochemical Sciences.

[bib20] Conkar D, Firat-Karalar EN (2021). Microtubule-associated proteins and emerging links to primary cilium structure, assembly, maintenance, and disassembly. The FEBS Journal.

[bib21] Consolati T, Locke J, Roostalu J, Chen ZA, Gannon J, Asthana J, Lim WM, Martino F, Cvetkovic MA, Rappsilber J, Costa A, Surrey T (2020). Microtubule nucleation properties of single human γturcs explained by their Cryo-EM structure. Developmental Cell.

[bib22] Conway L, Gramlich MW, Ali Tabei SM, Ross JL (2014). Microtubule orientation and spacing within bundles is critical for long-range kinesin-1 motility. Cytoskeleton.

[bib23] Coombes C, Yamamoto A, McClellan M, Reid TA, Plooster M, Luxton GW, Alper J, Howard J, Gardner MK (2016). Mechanism of microtubule lumen entry for the α-tubulin acetyltransferase enzyme αtat1. PNAS.

[bib24] Cunha-Ferreira I, Chazeau A, Buijs RR, Stucchi R, Will L, Pan X, Adolfs Y, van der Meer C, Wolthuis JC, Kahn OI, Schätzle P, Altelaar M, Pasterkamp RJ, Kapitein LC, Hoogenraad CC (2018). The HAUS complex is a key regulator of Non-centrosomal microtubule organization during neuronal development. Cell Reports.

[bib25] David AF, Roudot P, Legant WR, Betzig E, Danuser G, Gerlich DW (2019). Augmin accumulation on long-lived microtubules drives amplification and kinetochore-directed growth. Journal of Cell Biology.

[bib26] Davis LJ, Odde DJ, Block SM, Gross SP (2002). The importance of lattice defects in katanin-mediated microtubule severing in vitro. Biophysical Journal.

[bib27] de Forges H, Pilon A, Cantaloube I, Pallandre A, Haghiri-Gosnet AM, Perez F, Poüs C (2016). Localized mechanical stress promotes microtubule rescue. Current Biology.

[bib28] Deinum EE, Tindemans SH, Lindeboom JJ, Mulder BM (2017). How selective severing by Katanin promotes order in the plant cortical microtubule array. PNAS.

[bib29] Díaz-Valencia JD, Morelli MM, Bailey M, Zhang D, Sharp DJ, Ross JL (2011). *Drosophila* katanin-60 depolymerizes and severs at Microtubule defects. Biophysical Journal.

[bib30] Dimitrov A, Quesnoit M, Moutel S, Cantaloube I, Poüs C, Perez F (2008). Detection of GTP-tubulin conformation in vivo reveals a role for GTP remnants in microtubule rescues. Science.

[bib31] Dixit R, Chang E, Cyr R (2006). Establishment of polarity during organization of the acentrosomal plant cortical microtubule array. Molecular Biology of the Cell.

[bib32] Dixit R, Cyr R (2004). Encounters between dynamic cortical microtubules promote ordering of the cortical array through angle-dependent modifications of microtubule behavior. The Plant Cell.

[bib33] Dogterom M, Koenderink GH (2019). Actin-microtubule crosstalk in cell biology. Nature Reviews Molecular Cell Biology.

[bib34] Doodhi H, Katrukha EA, Kapitein LC, Akhmanova A (2014). Mechanical and geometrical constraints control kinesin-based microtubule guidance. Current Biology.

[bib35] Dumont EL, Do C, Hess H (2015). Molecular wear of microtubules propelled by surface-adhered kinesins. Nature Nanotechnology.

[bib36] Edozie B, Sahu S, Pitta M, Englert A, do Rosario CF, Ross JL (2019). Self-organization of spindle-like microtubule structures. Soft Matter.

[bib37] Eggert US, Mitchison TJ, Field CM (2006). Animal cytokinesis: from parts list to mechanisms. Annual Review of Biochemistry.

[bib38] Ehrhardt DW, Shaw SL (2006). Microtubule dynamics and organization in the plant cortical array. Annual Review of Plant Biology.

[bib39] Elliott A, Shaw SL (2018). Update: plant cortical microtubule arrays. Plant Physiology.

[bib40] Fink G, Szewczak-Harris A, Löwe J (2016). SnapShot: the bacterial cytoskeleton. Cell.

[bib41] Folker ES, Baker BM, Goodson HV (2005). Interactions between CLIP-170, tubulin, and microtubules: implications for the mechanism of Clip-170 plus-end tracking behavior. Molecular Biology of the Cell.

[bib42] Forth S, Kapoor TM (2017). The mechanics of microtubule networks in cell division. Journal of Cell Biology.

[bib43] Gadadhar S, Bodakuntla S, Natarajan K, Janke C (2017). The tubulin code at a glance. Journal of Cell Science.

[bib44] Gardiner J (2013). The evolution and diversification of plant microtubule-associated proteins. The Plant Journal.

[bib45] Gardner MK, Bouck DC, Paliulis LV, Meehl JB, O'Toole ET, Haase J, Soubry A, Joglekar AP, Winey M, Salmon ED, Bloom K, Odde DJ (2008). Chromosome congression by Kinesin-5 motor-mediated disassembly of longer kinetochore microtubules. Cell.

[bib46] Gaska I, Armstrong ME, Alfieri A, Forth S (2020). The mitotic crosslinking protein PRC1 acts like a mechanical dashpot to resist microtubule sliding. Developmental Cell.

[bib47] Gerson-Gurwitz A, Thiede C, Movshovich N, Fridman V, Podolskaya M, Danieli T, Lakämper S, Klopfenstein DR, Schmidt CF, Gheber L (2011). Directionality of individual kinesin-5 Cin8 motors is modulated by loop 8, ionic strength and microtubule geometry. The EMBO Journal.

[bib48] Glotzer M (2009). The 3ms of central spindle assembly: microtubules, motors and MAPs. Nature Reviews Molecular Cell Biology.

[bib49] Goshima G, Mayer M, Zhang N, Stuurman N, Vale RD (2008). Augmin: a protein complex required for centrosome-independent microtubule generation within the spindle. Journal of Cell Biology.

[bib50] Grover R, Fischer J, Schwarz FW, Walter WJ, Schwille P, Diez S (2016). Transport efficiency of membrane-anchored kinesin-1 motors depends on motor density and diffusivity. PNAS.

[bib51] Guo Y, Liu Y, Oldenbourg R, Tang JX, Valles JM (2008). Effects of osmotic force and torque on microtubule bundling and pattern formation. Physical Review E.

[bib52] Gupta ML, Carvalho P, Roof DM, Pellman D (2006). Plus end-specific depolymerase activity of Kip3, a kinesin-8 protein, explains its role in positioning the yeast mitotic spindle. Nature Cell Biology.

[bib53] Hannabuss J, Lera-Ramirez M, Cade NI, Fourniol FJ, Nédélec F, Surrey T (2019). Self-Organization of minimal anaphase spindle midzone bundles. Current Biology.

[bib54] Hibbel A, Bogdanova A, Mahamdeh M, Jannasch A, Storch M, Schäffer E, Liakopoulos D, Howard J (2015). Kinesin Kip2 enhances microtubule growth in vitro through length-dependent feedback on polymerization and catastrophe. eLife.

[bib55] Hitt AL, Cross AR, Williams RC (1990). Microtubule solutions display nematic liquid crystalline structure. Journal of Biological Chemistry.

[bib56] Hookway C, Ding L, Davidson MW, Rappoport JZ, Danuser G, Gelfand VI (2015). Microtubule-dependent transport and dynamics of vimentin intermediate filaments. Molecular Biology of the Cell.

[bib57] Howard J, Hudspeth AJ, Vale RD (1989). Movement of microtubules by single kinesin molecules. Nature.

[bib58] Howard J, Hyman AA (2007). Microtubule polymerases and depolymerases. Current Opinion in Cell Biology.

[bib59] Hunt AJ, Gittes F, Howard J (1994). The force exerted by a single kinesin molecule against a viscous load. Biophysical Journal.

[bib60] Janke C, Kneussel M (2010). Tubulin post-translational modifications: encoding functions on the neuronal microtubule cytoskeleton. Trends in Neurosciences.

[bib61] Janke C, Magiera MM (2020). The tubulin code and its role in controlling microtubule properties and functions. Nature Reviews Molecular Cell Biology.

[bib62] Janson ME, de Dood ME, Dogterom M (2003). Dynamic instability of microtubules is regulated by force. Journal of Cell Biology.

[bib63] Janson ME, Setty TG, Paoletti A, Tran PT (2005). Efficient formation of bipolar microtubule bundles requires microtubule-bound gamma-tubulin complexes. Journal of Cell Biology.

[bib64] Janson ME, Loughlin R, Loïodice I, Fu C, Brunner D, Nédélec FJ, Tran PT (2007). Crosslinkers and motors organize dynamic microtubules to form stable bipolar arrays in fission yeast. Cell.

[bib65] Kapitein LC, Kwok BH, Weinger JS, Schmidt CF, Kapoor TM, Peterman EJ (2008). Microtubule cross-linking triggers the directional motility of kinesin-5. Journal of Cell Biology.

[bib66] Kapitein LC, Hoogenraad CC (2015). Building the neuronal microtubule cytoskeleton. Neuron.

[bib67] Karsenti E (2008). Self-organization in cell biology: a brief history. Nature Reviews Molecular Cell Biology.

[bib68] Kaul N, Soppina V, Verhey KJ (2014). Effects of α-tubulin K40 acetylation and detyrosination on kinesin-1 motility in a purified system. Biophysical Journal.

[bib69] Kelliher MT, Saunders HA, Wildonger J (2019). Microtubule control of functional architecture in neurons. Current Opinion in Neurobiology.

[bib70] Khetan N, Pruliere G, Hebras C, Chenevert J, Athale CA (2021). Self-organized optimal packing of kinesin-5-driven microtubule asters scales with cell size. Journal of Cell Science.

[bib71] King MR, Petry S (2020). Phase separation of TPX2 enhances and spatially coordinates microtubule nucleation. Nature Communications.

[bib72] Kollman JM, Zelter A, Muller EG, Fox B, Rice LM, Davis TN, Agard DA (2008). The structure of the gamma-tubulin small complex: implications of its architecture and flexibility for microtubule nucleation. Molecular Biology of the Cell.

[bib73] Kong Z, Hotta T, Lee YR, Horio T, Liu B (2010). The {gamma}-tubulin complex protein GCP4 is required for organizing functional microtubule arrays in *Arabidopsis thaliana*. The Plant Cell.

[bib74] Krüger LK, Sanchez JL, Paoletti A, Tran PT (2019). Kinesin-6 regulates cell-size-dependent spindle elongation velocity to keep mitosis duration constant in fission yeast. eLife.

[bib75] Kuo YW, Trottier O, Mahamdeh M, Howard J (2019). Spastin is a dual-function enzyme that severs microtubules and promotes their regrowth to increase the number and mass of microtubules. PNAS.

[bib76] Leary A, Sim S, Nazarova E, Shulist K, Genthial R, Yang SK, Bui KH, Francois P, Vogel J (2019). Successive Kinesin-5 microtubule crosslinking and sliding promote fast, irreversible formation of a stereotyped bipolar spindle. Current Biology.

[bib77] Leduc C, Padberg-Gehle K, Varga V, Helbing D, Diez S, Howard J (2012). Molecular crowding creates traffic jams of kinesin motors on microtubules. PNAS.

[bib78] Lee YJ, Liu B (2019). Microtubule nucleation for the assembly of acentrosomal microtubule arrays in plant cells. New Phytologist.

[bib79] Leo L, Yu W, D'Rozario M, Waddell EA, Marenda DR, Baird MA, Davidson MW, Zhou B, Wu B, Baker L, Sharp DJ, Baas PW (2015). Vertebrate fidgetin restrains axonal growth by severing labile domains of microtubules. Cell Reports.

[bib80] Lera-Ramirez M, Nédélec FJ (2019). Theory of antiparallel microtubule overlap stabilization by motors and diffusible crosslinkers. Cytoskeleton.

[bib81] Lin Z, Gasic I, Chandrasekaran V, Peters N, Shao S, Mitchison TJ, Hegde RS (2020). TTC5 mediates autoregulation of tubulin via mRNA degradation. Science.

[bib82] Lindeboom JJ, Nakamura M, Hibbel A, Shundyak K, Gutierrez R, Ketelaar T, Emons AM, Mulder BM, Kirik V, Ehrhardt DW (2013). A mechanism for reorientation of cortical microtubule arrays driven by microtubule severing. Science.

[bib83] Liu T, Tian J, Wang G, Yu Y, Wang C, Ma Y, Zhang X, Xia G, Liu B, Kong Z (2014). Augmin triggers microtubule-dependent microtubule nucleation in interphase plant cells. Current Biology.

[bib84] Liu P, Zupa E, Neuner A, Böhler A, Loerke J, Flemming D, Ruppert T, Rudack T, Peter C, Spahn C, Gruss OJ, Pfeffer S, Schiebel E (2020). Insights into the assembly and activation of the microtubule nucleator γ-TuRC. Nature.

[bib85] Liu P, Würtz M, Zupa E, Pfeffer S, Schiebel E (2021). Microtubule nucleation: the waltz between γ-tubulin ring complex and associated proteins. Current Opinion in Cell Biology.

[bib86] Logan CM, Menko AS (2019). Microtubules: evolving roles and critical cellular interactions. Experimental Biology and Medicine.

[bib87] Lüders J (2021). Nucleating microtubules in neurons: challenges and solutions. Developmental Neurobiology.

[bib88] Lüders J, Stearns T (2007). Microtubule-organizing centres: a re-evaluation. Nature Reviews Molecular Cell Biology.

[bib89] Mastronarde DN, McDonald KL, Ding R, McIntosh JR (1993). Interpolar spindle microtubules in PTK cells. Journal of Cell Biology.

[bib90] Mattie FJ, Stackpole MM, Stone MC, Clippard JR, Rudnick DA, Qiu Y, Tao J, Allender DL, Parmar M, Rolls MM (2010). Directed microtubule growth, +TIPs, and kinesin-2 are required for uniform microtubule polarity in dendrites. Current Biology.

[bib91] Mayr MI, Hümmer S, Bormann J, Grüner T, Adio S, Woehlke G, Mayer TU (2007). The human kinesin Kif18A is a motile microtubule depolymerase essential for chromosome congression. Current Biology.

[bib92] McNally K, Audhya A, Oegema K, McNally FJ (2006). Katanin controls mitotic and meiotic spindle length. Journal of Cell Biology.

[bib93] McNally FJ, Roll-Mecak A (2018). Microtubule-severing enzymes: from cellular functions to molecular mechanism. Journal of Cell Biology.

[bib94] Miller PM, Folkmann AW, Maia AR, Efimova N, Efimov A, Kaverina I (2009). Golgi-derived CLASP-dependent microtubules control golgi organization and polarized trafficking in motile cells. Nature Cell Biology.

[bib95] Mitchison T, Wühr M, Nguyen P, Ishihara K, Groen A, Field CM (2012). Growth, interaction, and positioning of microtubule asters in extremely large vertebrate embryo cells. Cytoskeleton.

[bib96] Mitchison T, Kirschner M (1984). Dynamic instability of microtubule growth. Nature.

[bib97] Molodtsov MI, Mieck C, Dobbelaere J, Dammermann A, Westermann S, Vaziri A (2016). A Force-Induced directional switch of a molecular motor enables parallel microtubule bundle formation. Cell.

[bib98] Murata T, Sonobe S, Baskin TI, Hyodo S, Hasezawa S, Nagata T, Horio T, Hasebe M (2005). Microtubule-dependent microtubule nucleation based on recruitment of gamma-tubulin in higher plants. Nature Cell Biology.

[bib99] Murphy SM, Preble AM, Patel UK, O'Connell KL, Dias DP, Moritz M, Agard D, Stults JT, Stearns T (2001). GCP5 and GCP6: two new members of the human gamma-tubulin complex. Molecular Biology of the Cell.

[bib100] Musa H, Orton C, Morrison EE, Peckham M (2003). Microtubule assembly in cultured myoblasts and myotubes following nocodazole induced microtubule depolymerisation. Journal of Muscle Research and Cell Motility.

[bib101] Muto E, Sakai H, Kaseda K (2005). Long-range cooperative binding of kinesin to a microtubule in the presence of ATP. Journal of Cell Biology.

[bib102] Nakamura M, Ehrhardt DW, Hashimoto T (2010). Microtubule and katanin-dependent dynamics of microtubule nucleation complexes in the acentrosomal Arabidopsis cortical array. Nature Cell Biology.

[bib103] Nakamura M, Lindeboom JJ, Saltini M, Mulder BM, Ehrhardt DW (2018). SPR2 protects minus ends to promote severing and reorientation of plant cortical microtubule arrays. Journal of Cell Biology.

[bib104] Nakamura M, Hashimoto T (2009). A mutation in the Arabidopsis gamma-tubulin-containing complex causes helical growth and abnormal microtubule branching. Journal of Cell Science.

[bib105] Needleman DJ, Ojeda-Lopez MA, Raviv U, Ewert K, Jones JB, Miller HP, Wilson L, Safinya CR (2004a). Synchrotron X-ray diffraction study of microtubules buckling and bundling under osmotic stress: a probe of interprotofilament interactions. Physical Review Letters.

[bib106] Needleman DJ, Ojeda-Lopez MA, Raviv U, Miller HP, Wilson L, Safinya CR (2004b). Higher-order assembly of microtubules by counterions: from hexagonal bundles to living necklaces. PNAS.

[bib107] Needleman D, Dogic Z (2017). Active matter at the interface between materials science and cell biology. Nature Reviews Materials.

[bib108] Nixon FM, Gutiérrez-Caballero C, Hood FE, Booth DG, Prior IA, Royle SJ (2015). The mesh is a network of microtubule connectors that stabilizes individual kinetochore fibers of the mitotic spindle. eLife.

[bib109] Norris SR, Jung S, Singh P, Strothman CE, Erwin AL, Ohi MD, Zanic M, Ohi R (2018). Microtubule minus-end aster organization is driven by processive HSET-tubulin clusters. Nature communications.

[bib110] Noujaim M, Bechstedt S, Wieczorek M, Brouhard GJ (2014). Microtubules accelerate the kinase activity of Aurora-B by a reduction in dimensionality. PLOS ONE.

[bib111] O. WASTENEYS G, E. WILLIAMSON R (1989). Reassembly of microtubules in Nitella tasmanica: assembly of cortical microtubules in branching clusters and its relevance to steady-state microtubule assembly. Journal of Cell Science.

[bib112] Odde DJ, Ma L, Briggs AH, DeMarco A, Kirschner MW (1999). Microtubule bending and breaking in living fibroblast cells. Journal of Cell Science.

[bib113] Oegema K, Wiese C, Martin OC, Milligan RA, Iwamatsu A, Mitchison TJ, Zheng Y (1999). Characterization of two related *Drosophila* gamma-tubulin complexes that differ in their ability to nucleate microtubules. The Journal of Cell Biology.

[bib114] Oh D, Yu CH, Needleman DJ (2016). Spatial organization of the Ran pathway by microtubules in mitosis. PNAS.

[bib115] Pamula MC, Carlini L, Forth S, Verma P, Suresh S, Legant WR, Khodjakov A, Betzig E, Kapoor TM (2019). High-resolution imaging reveals how the spindle midzone impacts chromosome movement. Journal of Cell Biology.

[bib116] Paredez AR, Somerville CR, Ehrhardt DW (2006). Visualization of cellulose synthase demonstrates functional association with microtubules. Science.

[bib117] Paz J, Lüders J (2018). Microtubule-Organizing Centers: Towards a Minimal Parts List. Trends in Cell Biology.

[bib118] Peet DR, Burroughs NJ, Cross RA (2018). Kinesin expands and stabilizes the GDP-microtubule lattice. Nature Nanotechnology.

[bib119] Peterman EJ, Scholey JM (2009). Mitotic microtubule crosslinkers: insights from mechanistic studies. Current Biology: CB.

[bib120] Petry S, Groen AC, Ishihara K, Mitchison TJ, Vale RD (2013). Branching microtubule nucleation in *Xenopus* egg extracts mediated by augmin and TPX2. Cell.

[bib121] Petry S, Vale RD (2015). Microtubule nucleation at the centrosome and beyond. Nature Cell Biology.

[bib122] Pollard TD, O'Shaughnessy B (2019). Molecular Mechanism of Cytokinesis. Annual Review of Biochemistry.

[bib123] Portran D, Schaedel L, Xu Z, Théry M, Nachury MV (2017). Tubulin acetylation protects long-lived microtubules against mechanical ageing. Nature Cell Biology.

[bib124] Pringle J, Muthukumar A, Tan A, Crankshaw L, Conway L, Ross JL (2013). Microtubule organization by kinesin motors and microtubule crosslinking protein MAP65. Journal of Physics: Condensed Matter.

[bib125] Rai A, Liu T, Glauser S, Katrukha EA, Estévez-Gallego J, Rodríguez-García R, Fang WS, Díaz JF, Steinmetz MO, Altmann KH, Kapitein LC, Moores CA, Akhmanova A (2020). Taxanes convert regions of perturbed microtubule growth into rescue sites. Nature Materials.

[bib126] Rai A, Liu T, Katrukha EA, Estévez-Gallego J, Paterson I, Diaz F, Kapitein LC, Moores CA, Akhmanova A (2021). Microtubule lattice defects promote catastrophes. bioRxiv.

[bib127] Rickman J, Nédélec F, Surrey T (2019). Effects of spatial dimensionality and steric interactions on microtubule-motor self-organization. Physical Biology.

[bib128] Rizk RS, Discipio KA, Proudfoot KG, Gupta ML (2014). The kinesin-8 Kip3 scales anaphase spindle length by suppression of midzone microtubule polymerization. The Journal of Cell Biology.

[bib129] Robison P, Caporizzo MA, Ahmadzadeh H, Bogush AI, Chen CY, Margulies KB, Shenoy VB, Prosser BL (2016). Detyrosinated microtubules buckle and bear load in contracting cardiomyocytes. Science.

[bib130] Roll-Mecak A, Vale RD (2006). Making more microtubules by severing: a common theme of noncentrosomal microtubule arrays?. The Journal of Cell Biology.

[bib131] Roostalu J, Hentrich C, Bieling P, Telley IA, Schiebel E, Surrey T (2011). Directional switching of the kinesin Cin8 through motor coupling. Science.

[bib132] Roostalu J, Rickman J, Thomas C, Nédélec F, Surrey T (2018). Determinants of Polar versus Nematic Organization in Networks of Dynamic Microtubules and Mitotic Motors. Cell.

[bib133] Roostalu J, Surrey T (2017). Microtubule nucleation: beyond the template. Nature Reviews. Molecular Cell Biology.

[bib134] Sánchez I, Dynlacht BD (2016). Cilium assembly and disassembly. Nature Cell Biology.

[bib135] Sánchez-Huertas C, Freixo F, Viais R, Lacasa C, Soriano E, Lüders J (2016). Non-centrosomal nucleation mediated by augmin organizes microtubules in post-mitotic neurons and controls axonal microtubule polarity. Nature Communications.

[bib136] Schaedel L, John K, Gaillard J, Nachury MV, Blanchoin L, Théry M (2015). Microtubules self-repair in response to mechanical stress. Nature Materials.

[bib137] Schaedel L, Triclin S, Chrétien D, Abrieu A, Aumeier C, Gaillard J, Blanchoin L, Théry M, John K (2019). Lattice defects induce microtubule self-renewal. Nature Physics.

[bib138] Schmidt-Cernohorska M, Zhernov I, Steib E, Le Guennec M, Achek R, Borgers S, Demurtas D, Mouawad L, Lansky Z, Hamel V, Guichard P (2019). Flagellar microtubule doublet assembly in vitro reveals a regulatory role of tubulin C-terminal tails. Science.

[bib139] Scholey J, Civelekoglu-Scholey G, Brust-Mascher I (2016). Anaphase B. Biology.

[bib140] Schwarz PM, Liggins JR, Ludueña RF (1998). Beta-tubulin isotypes purified from bovine brain have different relative stabilities. Biochemistry.

[bib141] Seetharaman S, Etienne-Manneville S (2020). Cytoskeletal Crosstalk in Cell Migration. Trends in Cell Biology.

[bib142] Serrano L, de la Torre J, Maccioni RB, Avila J (1984). Involvement of the carboxyl-terminal domain of tubulin in the regulation of its assembly. PNAS.

[bib143] She ZY, Wei YL, Lin Y, Li YL, Lu MH (2019). Mechanisms of the Ase1/PRC1/MAP65 family in central spindle assembly. Biological Reviews of the Cambridge Philosophical Society.

[bib144] Shima T, Morikawa M, Kaneshiro J, Kambara T, Kamimura S, Yagi T, Iwamoto H, Uemura S, Shigematsu H, Shirouzu M, Ichimura T, Watanabe TM, Nitta R, Okada Y, Hirokawa N (2018). Kinesin-binding-triggered conformation switching of microtubules contributes to polarized transport. The Journal of Cell Biology.

[bib145] Shimamoto Y, Forth S, Kapoor TM (2015). Measuring Pushing and Braking Forces Generated by Ensembles of Kinesin-5 Crosslinking Two Microtubules. Developmental Cell.

[bib146] Sirajuddin M, Rice LM, Vale RD (2014). Regulation of microtubule motors by tubulin isotypes and post-translational modifications. Nature Cell Biology.

[bib147] Song JG, King MR, Zhang R, Kadzik RS, Thawani A, Petry S (2018). Mechanism of how augmin directly targets the γ-tubulin ring complex to microtubules. The Journal of Cell Biology.

[bib148] Song Y, Brady ST (2015). Post-translational modifications of tubulin: pathways to functional diversity of microtubules. Trends in Cell Biology.

[bib149] Srayko M, O'toole ET, Hyman AA, Müller-Reichert T (2006). Katanin disrupts the microtubule lattice and increases polymer number in *C. elegans* meiosis. Current Biology: CB.

[bib150] Stairs CW, Ettema TJG (2020). The Archaeal Roots of the Eukaryotic Dynamic Actin Cytoskeleton. Current Biology: CB.

[bib151] Stanhope KT, Yadav V, Santangelo CD, Ross JL (2017). Contractility in an extensile system. Soft Matter.

[bib152] Stone MC, Roegiers F, Rolls MM (2008). Microtubules have opposite orientation in axons and dendrites of *Drosophila* neurons. Molecular Biology of the Cell.

[bib153] Subramanian R, Ti SC, Tan L, Darst SA, Kapoor TM (2013). Marking and measuring single microtubules by PRC1 and kinesin-4. Cell.

[bib154] Subramanian R, Kapoor TM (2012). Building complexity: insights into self-organized assembly of microtubule-based architectures. Developmental Cell.

[bib155] Szyk A, Deaconescu AM, Piszczek G, Roll-Mecak A (2011). Tubulin tyrosine ligase structure reveals adaptation of an ancient fold to bind and modify tubulin. Nature Structural & Molecular Biology.

[bib156] Szyk A, Deaconescu AM, Spector J, Goodman B, Valenstein ML, Ziolkowska NE, Kormendi V, Grigorieff N, Roll-Mecak A (2014). Molecular basis for age-dependent microtubule acetylation by tubulin acetyltransferase. Cell.

[bib157] Tan R, Foster PJ, Needleman DJ, McKenney RJ (2018). Cooperative Accumulation of Dynein-Dynactin at Microtubule Minus-Ends Drives Microtubule Network Reorganization. Developmental Cell.

[bib158] Tariq A, Green L, Jeynes JCG, Soeller C, Wakefield JG (2020). In vitro reconstitution of branching microtubule nucleation. eLife.

[bib159] Tian J, Kong Z (2019). The role of the augmin complex in establishing microtubule arrays. Journal of Experimental Botany.

[bib160] Tropini C, Roth EA, Zanic M, Gardner MK, Howard J (2012). Islands containing slowly hydrolyzable GTP analogs promote microtubule rescues. PLOS ONE.

[bib161] Uehara R, Kamasaki T, Hiruma S, Poser I, Yoda K, Yajima J, Gerlich DW, Goshima G (2016). Augmin shapes the anaphase spindle for efficient cytokinetic furrow ingression and abscission. Molecular Biology of the Cell.

[bib162] Uehara R, Goshima G (2010). Functional central spindle assembly requires de novo microtubule generation in the interchromosomal region during anaphase. The Journal of Cell Biology.

[bib163] Valenstein ML, Roll-Mecak A (2016). Graded Control of Microtubule Severing by Tubulin Glutamylation. Cell.

[bib164] van Beuningen SF, Hoogenraad CC (2016). Neuronal polarity: remodeling microtubule organization. Current Opinion in Neurobiology.

[bib165] van de Willige D, Hoogenraad CC, Akhmanova A (2016). Microtubule plus-end tracking proteins in neuronal development. Cellular and Molecular Life Sciences.

[bib166] Varga V, Helenius J, Tanaka K, Hyman AA, Tanaka TU, Howard J (2006). Yeast kinesin-8 depolymerizes microtubules in a length-dependent manner. Nature Cell Biology.

[bib167] Varga V, Leduc C, Bormuth V, Diez S, Howard J (2009). Kinesin-8 motors act cooperatively to mediate length-dependent microtubule depolymerization. Cell.

[bib168] Vemu A, Szczesna E, Zehr EA, Spector JO, Grigorieff N, Deaconescu AM, Roll-Mecak A (2018). Severing enzymes amplify microtubule arrays through lattice GTP-tubulin incorporation. Science.

[bib169] Verhey KJ, Gaertig J (2007). The tubulin code. Cell Cycle.

[bib170] Vilfan A, Frey E, Schwabl F, Thormählen M, Song YH, Mandelkow E (2001). Dynamics and cooperativity of microtubule decoration by the motor protein kinesin. Journal of Molecular Biology.

[bib171] Vukušić K, Buđa R, Tolić IM (2019). Force-generating mechanisms of anaphase in human cells. Journal of Cell Science.

[bib172] Walker RA, O'Brien ET, Pryer NK, Soboeiro MF, Voter WA, Erickson HP, Salmon ED (1988). Dynamic instability of individual microtubules analyzed by video light microscopy: rate constants and transition frequencies. The Journal of Cell Biology.

[bib173] Wang C, Liu W, Wang G, Li J, Dong L, Han L, Wang Q, Tian J, Yu Y, Gao C, Kong Z (2017). KTN80 confers precision to microtubule severing by specific targeting of katanin complexes in plant cells. The EMBO Journal.

[bib174] Wasteneys GO, Ambrose JC (2009). Spatial organization of plant cortical microtubules: close encounters of the 2D kind. Trends in Cell Biology.

[bib175] Watanabe T, Noritake J, Kaibuchi K (2005). Regulation of microtubules in cell migration. Trends in Cell Biology.

[bib176] Waterman-Storer CM, Salmon ED (1997). Actomyosin-based retrograde flow of microtubules in the lamella of migrating epithelial cells influences microtubule dynamic instability and turnover and is associated with microtubule breakage and treadmilling. The Journal of Cell Biology.

[bib177] Wieczorek M, Urnavicius L, Ti S-C, Molloy KR, Chait BT, Kapoor TM (2020). Asymmetric molecular architecture of the human γ-Tubulin ring complex. Cell.

[bib178] Wightman R, Turner SR (2007). Severing at sites of microtubule crossover contributes to microtubule alignment in cortical arrays. The Plant Journal.

[bib179] Wijeratne SS, Fiorenza SA, Subramanian R, Betterton MD (2020). Motor guidance by long-range communication through the microtubule highway. bioRxiv.

[bib180] Wijeratne S, Subramanian R (2018). Geometry of antiparallel microtubule bundles regulates relative sliding and stalling by PRC1 and Kif4A. eLife.

[bib181] Wloga D, Joachimiak E, Louka P, Gaertig J (2017). Posttranslational modifications of tubulin and cilia. Cold Spring Harbor Perspectives in Biology.

[bib182] Wu J, Akhmanova A (2017). Microtubule-Organizing centers. Annual Review of Cell and Developmental Biology.

[bib183] Yagi N, Matsunaga S, Hashimoto T (2018). Insights into cortical microtubule nucleation and dynamics in *Arabidopsis* leaf cells. Journal of Cell Science.

[bib184] Yi P, Goshima G (2018). Microtubule nucleation and organization without centrosomes. Current Opinion in Plant Biology.

[bib185] Zandomeni K, Schopfer P (1993). Reorientation of microtubules at the outer epidermal wall of maize coleoptiles by Phytochrome, blue-light photoreceptor, and auxin. Protoplasma.

[bib186] Zanic M, Widlund PO, Hyman AA, Howard J (2013). Synergy between XMAP215 and EB1 increases microtubule growth rates to physiological levels. Nature Cell Biology.

[bib187] Zhang Q, Fishel E, Bertroche T, Dixit R (2013). Microtubule severing at crossover sites by katanin generates ordered cortical microtubule arrays in Arabidopsis. Current Biology: CB.

[bib188] Zheng Y, Wong ML, Alberts B, Mitchison T (1995). Nucleation of microtubule assembly by a gamma-tubulin-containing ring complex. Nature.

